# Assessing urban strategies for reducing the impacts of extreme weather on infrastructure networks

**DOI:** 10.1098/rsos.160023

**Published:** 2016-05-11

**Authors:** Maria Pregnolato, Alistair Ford, Craig Robson, Vassilis Glenis, Stuart Barr, Richard Dawson

**Affiliations:** School of Civil Engineering and Geosciences, Newcastle University, Newcastle upon Tyne, UK

**Keywords:** network, flooding, impact, modelling, transport

## Abstract

Critical infrastructure networks, including transport, are crucial to the social and economic function of urban areas but are at increasing risk from natural hazards. Minimizing disruption to these networks should form part of a strategy to increase urban resilience. A framework for assessing the disruption from flood events to transport systems is presented that couples a high-resolution urban flood model with transport modelling and network analytics to assess the impacts of extreme rainfall events, and to quantify the resilience value of different adaptation options. A case study in Newcastle upon Tyne in the UK shows that both green roof infrastructure and traditional engineering interventions such as culverts or flood walls can reduce transport disruption from flooding. The magnitude of these benefits depends on the flood event and adaptation strategy, but for the scenarios considered here 3–22% improvements in city-wide travel times are achieved. The network metric of betweenness centrality, weighted by travel time, is shown to provide a rapid approach to identify and prioritize the most critical locations for flood risk management intervention. Protecting just the top ranked critical location from flooding provides an 11% reduction in person delays. A city-wide deployment of green roofs achieves a 26% reduction, and although key routes still flood, the benefits of this strategy are more evenly distributed across the transport network as flood depths are reduced across the model domain. Both options should form part of an urban flood risk management strategy, but this method can be used to optimize investment and target limited resources at critical locations, enabling green infrastructure strategies to be gradually implemented over the longer term to provide city-wide benefits. This framework provides a means of prioritizing limited financial resources to improve resilience. This is particularly important as flood management investments must typically exceed a far higher benefit–cost threshold than transport infrastructure investments. By capturing the value to the transport network from flood management interventions, it is possible to create new business models that provide benefits to, and enhance the resilience of, both transport and flood risk management infrastructures. Further work will develop the framework to consider other hazards and infrastructure networks.

## 1. Introduction

An increasing number of catastrophic weather-related events across the globe have seen the safety and resilience of infrastructure networks become an important issue in recent years. In Europe, 40 major catastrophic events took place in 2013, in particular two of the most costly in the world ($16.5 billion of damage across Germany and Czech Republic from flooding; $4.8 billion in France and Germany caused by hailstorms), according to Swiss RE [1]. The UK has also suffered multiple extreme weather events, such as the 2007 floods in England (£3.2 billion economic losses) and the 2009 Cumbria floods (£276 million) [2]. The expected effects of climate change, in particular shifts in weather patterns with drier summers, rising sea-levels, wetter winters and flash floods, will mean that conditions are expected to worsen in the future [3–6]. Concurrently, an increase in population and economic activities in urban areas will place further pressure on infrastructure systems [7]. While this concentration of assets and activity drives productivity and creativity in cities, it also increases their exposure to hazards. Infrastructure networks play a crucial role in mediating both the risks and opportunities to people and the economy but must be reconfigured as part of a strategy to increase resilience in urban environments [8–11].

This paper introduces an integrated assessment framework to quantify the impact of flooding on disruption to road transport, which has been poorly studied [12]. The framework couples high-resolution flood modelling with a transport network model to assess the impact of different flood events on transport disruption, and to assess the benefits of flood risk management measures in terms of transport disruption. These are compared with network betweenness centrality (BC) to explore the criticality of major roads and junctions/intersections of the network under a hazard event and to identify priority locations for intervention. Following this introductory section, further background about the problem is provided before introducing the methodology in §3 and subsequently a case study application in Newcastle upon Tyne in the UK. The implications of the results for flood risk and transport infrastructure managers are discussed, before drawing appropriate conclusions.

## 2. Background

### 2.1. Disruption to transport infrastructure

Disruption to critical infrastructure is of major interest to engineers, policy-makers and planners. A resilient transport system is considered to have the ability ‘to withstand the impacts of extreme weather, to operate in the face of such weather and to recover promptly from its effects' [13]. The focus of this paper is on the impacts and operation during extreme weather, rather than recovery. To improve the ability to avoid or limit the effects of hazard events (such as flooding) where possible, it is essential that a greater level of understanding of the behaviour of networks under hazard conditions is developed, alongside improved modelling methodologies and techniques which can allow the assessment of options for reducing the impact of hazards [14].

Transport has been recognized as particularly vulnerable to extreme events and climate change [15]. Flooding can be caused by groundwater, coastal, fluvial or pluvial events and is one of the most serious hazards globally due to the impacts it causes on the environment, social well-being and the economy. In many cities, intense rainfall coupled with inadequate, or poorly maintained, local drainage systems can lead to the rapid onset of surface water flooding causing damage to surface infrastructure and disruption to transport networks. The impact of this disruption can extend far beyond the flood extent due to the connectivity of the transport system [16].

A number of recent studies have examined the impact of weather events on urban transportation; however, they focus on impacts to traffic speeds due to ice, snow, precipitation (that hampers driver visibility as opposed to flooding) and wind [3,12,15,17–19]. Traffic safety and travel times for road transport have been investigated for many weather-related phenomena (e.g. fog, wind, rain, snow, ice), but flooding is generally missing from this literature [12], apart from analysis of water forces on parked vehicles [20,21]. Investigations into the impact of floods on road networks focused on road closures or car accidents without considering traffic speed and travel time [22–24].

### 2.2. Network analysis

Network complexity science has been used for the analysis of the resilience of critical infrastructure to understand the dynamics of failures within networked systems, often adopting purely topological or metric-based approaches to simulate the failure of components [25–27]. While useful, these approaches do not capture the physical properties, attributes and engineering failure mechanisms of real infrastructure components. Furthermore, the focus of these studies is typically on system-wide failure rather than considering the implications of reduced performance in part or all of the network (e.g. links becoming more difficult to traverse). As network models are typically aspatial, the emphasis has been on topological interactions rather than considering the geography of the hazard and infrastructure. This leads to the loss of critical information for improving the ability of these systems to withstand hazards, many of which vary geographically [28].

However, graph theory can provide some useful measures of network performance. For example, the relative importance of nodes and edges can be effectively obtained through centrality measures. Traditional models of network analysis, however, are often limited to topological and graph theory approaches, considering the network only as links and nodes, and ignoring flows and other features [29]. This paper considers the relationship between such network metrics and the impact of floods on transport flows. Recent events, such as the extreme rainfall event on 28 June 2012 in Newcastle upon Tyne in the northeast of England, have demonstrated the need to develop a greater understanding of extreme rainfall events, in order to maintain network performance at an acceptable level during extreme weather events.

### 2.3. Adaptation to flood risk

The adaptation of transport systems, to manage the impacts of extreme weather events, is not limited to improved design of transport infrastructure alone. Urban flood risk management can be implemented at the source of flooding (e.g. extreme rainfall causing a build-up of surface water), along its pathway (e.g. an overland flow of surface run-off) or at the receptor itself (e.g. a section of road in the transport network) [30,31].

Adaptation can involve moving infrastructure away from flood-prone areas (i.e. diverting roads), improving the resilience of existing infrastructure *in situ* (i.e. improving drainage along roads, raising roads where flooding is expected to occur), or use of permeable paving [32]. An increasingly important option for increasing urban resilience to flooding are sustainable urban drainage systems (SUDS). SUDS pay particular attention to the source and pathway stages of the flood process [33], as opposed to traditional engineering solutions to stormwater management (e.g. drainage channels and sewerage systems). SUDS seek to use natural processes to reduce initial run-off through source interventions, such as blue or green surfaces (e.g. parks, ponds, roofs), and to increase the retention and infiltration of water [34]. These adaptations intercept or reduce run-off before it reaches the receptors, thereby reducing the magnitude of the hazard at the infrastructure. Such measures are also often referred to as Blue Green Infrastructure (BGI). Other options include building redundancy into the network (i.e. providing alternative routes) or increasing mode share for more resilient transport modes (i.e. encouraging shift from private car-based transport to public transport, walking and cycling) [35].

The use of BGI to address the impacts of urban extreme weather events is of particular interest as BGI provides additional benefits beyond flood risk reduction. However, the evidence to support the use of BGI to manage extreme events is often ambiguous [36]. This study compares a traditional and BGI strategy to manage the impacts of flooding on transport disruption to understand how urban environments can be impacted by extreme rainfall events and which strategies could help to better protect them from present and future flooding. The emergence of new sources of data, for example, from ubiquitous sensors or from the exploitation of big data, and the advancement of network modelling techniques have increased the ability to simulate and understand ever-more complex urban processes [37]. The Tyne and Wear metropolitan area (UK) has been chosen for preliminary analysis due to the availability of such observations from recent extreme weather events.

## 3. Material and methods

**Figure 1.**
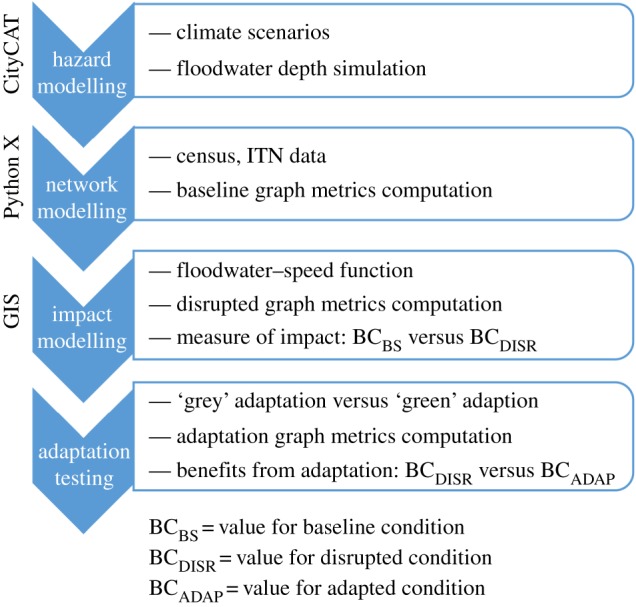
The integrated assessment framework for transport disruption analysis.

An integrated assessment framework that combines hazard modelling, graph theory and transport networks analysis is used to enable evaluation of the effectiveness of different adaptation strategies (figure 1). This work has used data and models that are appropriate to a UK application but, data permitting, the principles are transferable.

### 3.1. Flood hazard model

The impact of a flood depends on a number of factors, including water depth, water velocity, flood duration and the spatial extent of inundation. The focus here is on flooding from heavy rainfall. Flood hazard is quantified using the City Catchment Analysis Tool (CityCAT), a hydrodynamic model developed to simulate pluvial inundation at high resolution, to account for the complexity of the built environment [38]. CityCAT is a two-dimensional hydrodynamic flood model based on the evaluation of infiltration of pervious areas, and it produces temporal series of floodwater depths. In recent years, cloud computing has made possible the simulation of a large number of ensembles, allowing assessment of the uncertainty and variability of extreme rain events in the present and future conditions [39]. Simulations can be undertaken on current climatic conditions or future scenarios, based on the rainfall duration and return period, for large domains up to millions of computational cells. A digital terrain model (DTM) of all the catchment basins that flow into Newcastle upon Tyne was used as an input to the hazard model to generate the underlying topography. In this case, the UK Environment Agency's 4 m DTM is used with building footprints, green spaces and other urban structures which are incorporated from the UK Ordnance Survey MasterMap data. Inclusion of buildings, soil porosity and other characteristics allows more realistic simulation of flow paths in urban areas.

### 3.2. Flows over the transport network

The vulnerability of an infrastructure asset depends on both its role in the network (assessed by graph measures, see §3.3) and the number of users who rely on the asset during their use of the network. To estimate the number of people using a particular link in the network, and thus the number of people affected by a reduction in its performance, a macro-scale traffic model has been developed to simulate flows on urban transport networks under various hazard scenarios. Transport journeys between origin and destination locations (e.g. places of residence and employment) are estimated using a trip-assignment routine, which simulates commuting journeys along each segment of the road network [40]. Travel time is computed as a function of a number of attributes, i.e. distance, free-flow speed, capacity and congestion and for private transport road users. A matrix of peak traffic flows between origins and destinations for each mode is constructed from census and travel survey data. People trips are converted to vehicle trips using established values for vehicle occupancy (for example, a mean of 1.16 people per car for commuting trips is given in the UK Department for Transport's WebTAG guidance [41]).

A network is constructed from the Ordnance Survey ITN (Integrated Transport Network) data layer, a nationally available UK dataset specifically designed for network analysis (and thus supplied in a topologically correct form). Each link on the transport network is attributed, based upon its capacity, with a function that relates speed reductions to flow increases to capture the influence of congestion. Equilibrium is sought by travellers selecting routes that find the least cost path between origin O and destination D (as used to calculate BC, see §3.3) using the approach described by Ford *et al*. [42] in both normal, unperturbed conditions and following disruption due to a flooding hazard.

### 3.3. Betweenness centrality of transport flows

The BC metric of the topologically valid representation of the road network provides an effective indicator of a node's centrality in a given network, and is used to identify the most critical nodes/edges [43–45]. The BC represents the number of shortest paths which pass through it when considering the paths between each pair of nodes in the network. This is normalized by the total number of shortest paths in the network to give a centrality value [46–48], which acts as a proxy for flows through networks [43,45,49]. The BC for node *v* is [48]

3.1 $$C_{B}(v)=\underset{s,t\;\in \;V}{\sum }\frac{\sigma (s,\;\;t|v)}{\sigma (s,\;t)},\;$$

where *V* is the set of nodes which are in the network, *N* is the number of nodes in the network, *σ*(*s*,*t*) is number of shortest paths and *σ*(*s*,*t*|*v*) is the number of shortest paths which pass through node *v* other than *s,t*. Here, the shortest path is calculated in terms of travel time, rather than distance, in keeping with the traffic flow model described in §3.2. By weighting the BC in this way, the measure can be used to assess disruptive events, in particular where disruptions do not necessarily result in the complete failure of network components. Instead, where a disruption occurs, the weight on an edge increases to reflect slower travel speed (higher travel time), resulting in the shortest paths for the BC measure avoiding the affected edges where alternative routes are quicker. This allows the modelling of events where the scale of disruption varies between edges rather than simple Boolean failures associated with other graph theory studies. Furthermore, the metric also allows for the identification of the critical locations in the network based as it captures the locations of the greatest disruption to normal traffic behaviour as a result of a flood event. This calculation is undertaken using an open source software stack consisting of a postgreSQL relational database with the spatial extension postGIS which allows the explicit handling of spatial data, and through a developed network schema and wrapper the explicit handling of networks [50]. The NetworkX Python library, through the developed wrapper, is then used for the computational analysis of the network.

### 3.4. Assessing disruption and the benefits of adaptation

**Figure 2.**
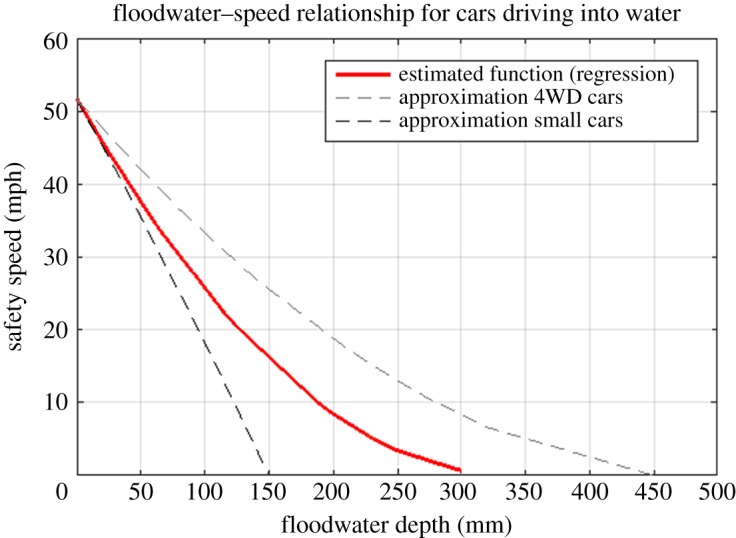
Depth-disruption function that relates vehicle speed and flood depth on a road.

To calculate the disruption to network links as a result of flooding impacts, timeseries of floodwater depths across the model domain are integrated with the spatial network model. Flood water reduces speeds, or stops entirely, traffic flows along flooded network links according to the depth of inundation. Existing methods (e.g. [24]) assume roads that are flooded to any depth to be entirely closed. This is perhaps suitable for fluvial or coastal inundation where depths are typically large across the flood extent, but flood depths from intense rainfall can vary substantially according to local conditions. To address this, Pregnolato *et al*. [51] developed a new curve that relates water depth (between 0 and a critical flood depth where the road is impassable) to safe driving car speed (figure 2). This function has been developed by combining data from experimental reports [52], safety literature [18,53,54], experimental data [55], analysis of videos of cars driving through floodwater and expert judgement (e.g. Automobile Association). The maximum threshold above which water becomes impassable (i.e. the average sill height of a normal motor car) is considered to be 30 cm for most cars; therefore, a link is assumed closed only when the limit of 30 cm is reached. An upper and lower confidence interval is considered, to include uncertainties due to driving characteristic and behaviour (e.g. type of car, asphalt or tyre, behaviour of the driver, visibility). The shape of the function is dependent upon the speed limit of the road; figure 2 shows the impact on a road with an initial speed limit of 50 mph.

**Figure 3.**
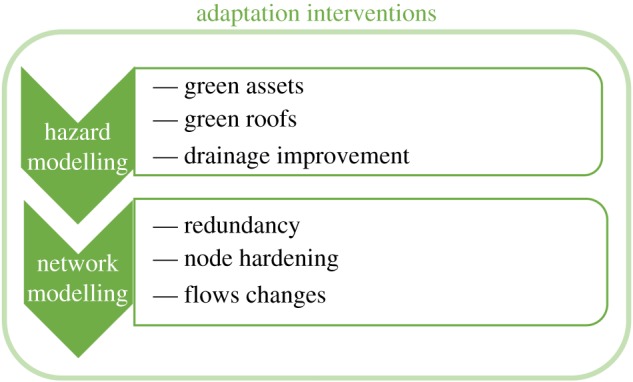
Implementation of adaptation intervention within the framework.

Thus, the network properties of a link (e.g. travel time) are modified according to this relationship, and transport flows (§3.2) and BC (§3.3) are recalculated for this perturbed state. Adaptation scenarios can be tested by altering inputs to either CityCAT or the transport network. As noted in §2.3, these include a combination of measures from hard measures of traditional engineering (e.g. drainage improvement) to ‘soft measures' of green infrastructure (e.g. rain gardens) which can be represented in the model by the modification of coefficients of infiltration and storage in CityCAT, or by adjusting the properties of links and nodes in the network model (figure 3).

The impact of flood events is assessed in terms of increases to the overall travel time, *T*_net_, across the network:

3.2 $$T_{\mathrm{net}}=\underset{i}{\sum }\underset{j}{\sum }t_{i,j},\;$$

where *t* is the journey time between zone *i* and zone *j*, and as a ‘person minute delay’, *T*_person_, that accounts for the traffic flow, *q*, between each zone:

3.3 $$T_{\mathrm{person}}=\underset{i}{\sum }\underset{j}{\sum }q_{i,j}t_{i,j},\;$$

where *Q* is the total volume of traffic across the network. Since trips have an associated cost (e.g. lost work-time, or reduction in productivity), the economic impacts could be calculated using the relationships described in WebTAG [41]. By comparing baseline and perturbed values of BC, an estimation of the impact of disruptions on the network behaviour is obtained.

## 4. Results

### 4.1. Newcastle upon Tyne (UK) case study

**Figure 4.**
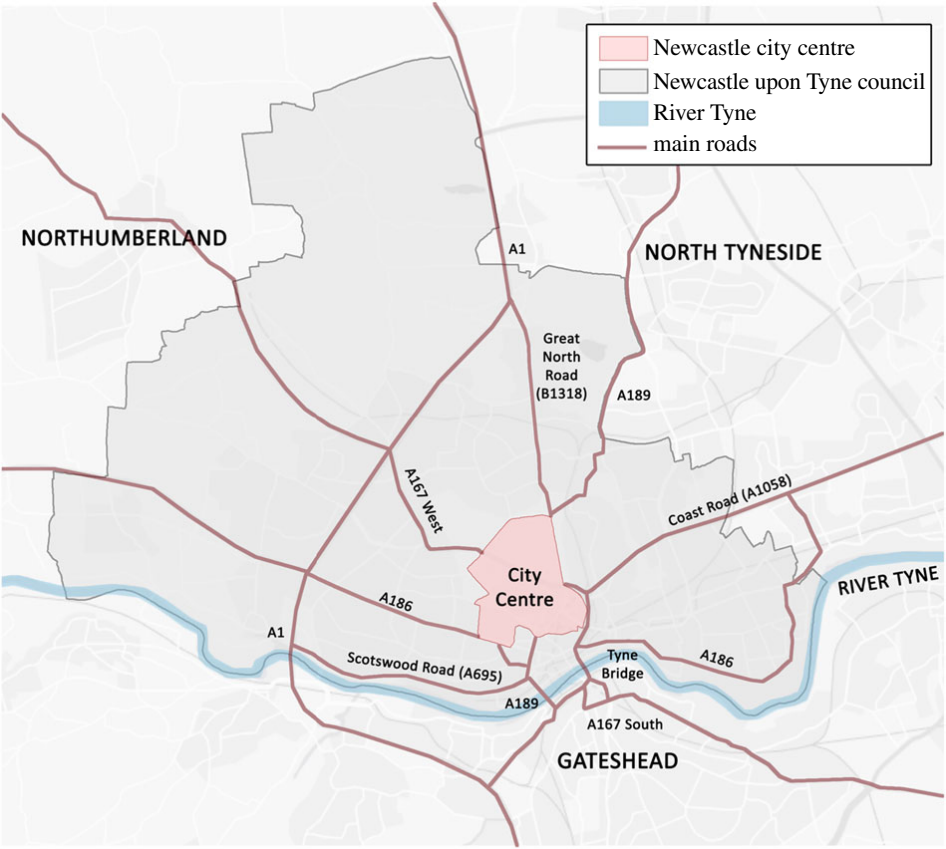
Newcastle upon Tyne and major roads in Tyne and Wear.

The framework has been applied to Newcastle upon Tyne in northeast England (figure 4). The city has been flooded by intense rainfall on previous occasions, and the city centre is almost impervious (92%) and without an overarching strategy for its drainage system it provides a useful prototype in the UK for the analysis of flash floods [56].

**Figure 5.**
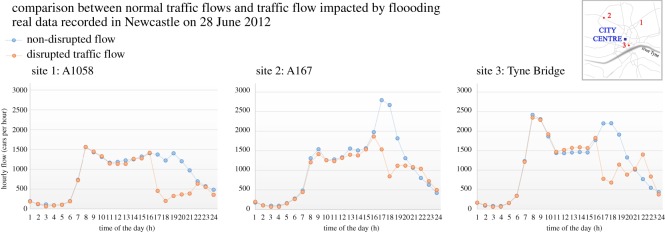
Comparison between the non-perturbed traffic flow (blue) and the disrupted flow (orange) of the extreme flood event on 28 June 2012, from Newcastle TADU traffic data (cars per hour).

Additionally, the presence of historical data in Newcastle, collected during past flood events by the Traffic Accident Data Unit (TADU), enables some validation. An example of recorded data, obtained through automatic traffic counters during the flood on 28 June 2012, is shown in figure 5. This event had an estimated return period of 1 in 100 years, and an approximate duration of 2 hours led to inundation of 377 road links, of which 227 suffered highway damage [57].

The transport model has been implemented for Newcastle upon Tyne using census data from 2011 Journey To Work records at Middle Super Output Area (MSOA) level. This provides the number of people travelling between each pair of MSOA zones and the mode of transport they take for their journey, which are aggregated using the method described in §3.2 to assign baseline traffic flows to the road network during peak commuting periods.

**Figure 6.**
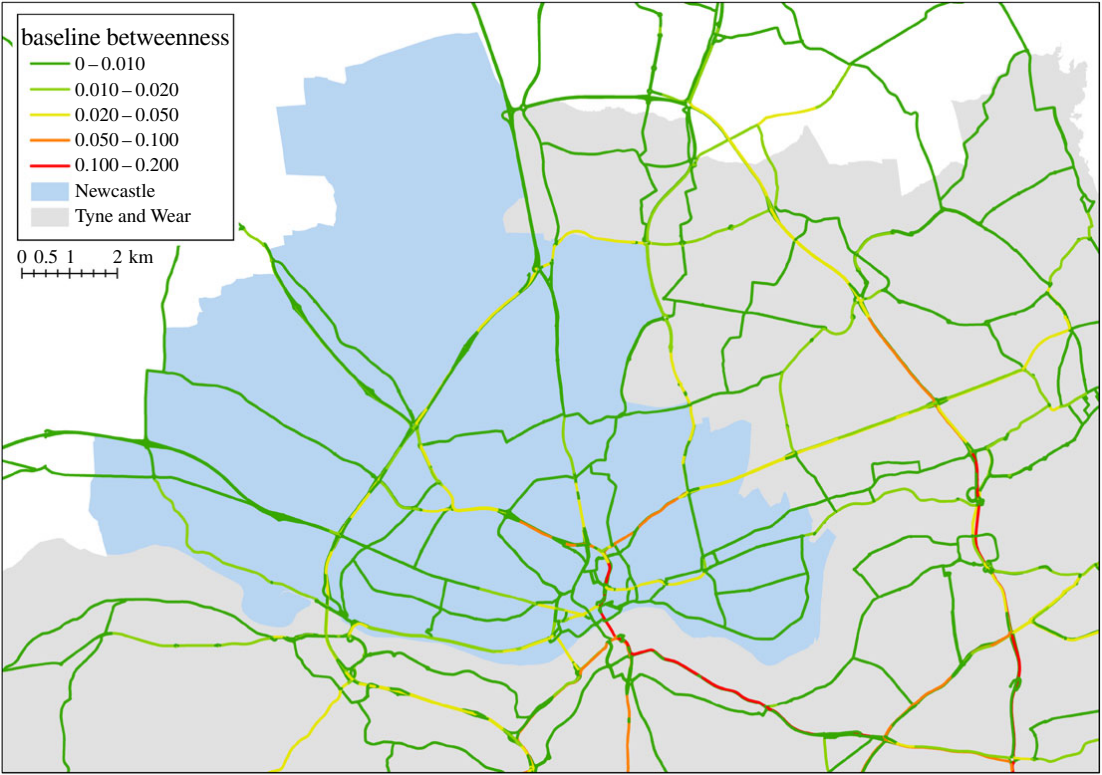
BC for the major roads in Newcastle and the wider Tyne and Wear region.

The BC is computed over the road network for the wider Tyne and Wear local authority of which the Newcastle City Council forms a significant part, weighted using the time to travel (figure 6). Critical features of this network, such as the limited river crossings to the south, along with the main roads into the centre of the Newcastle area, have some of the highest BC values. This provides a baseline set of values for all major roads in the area of interest from which the impacts of hazards on this critical urban system can be compared against and the sensitivity to applied adaptations analysed.

**Figure 7.**
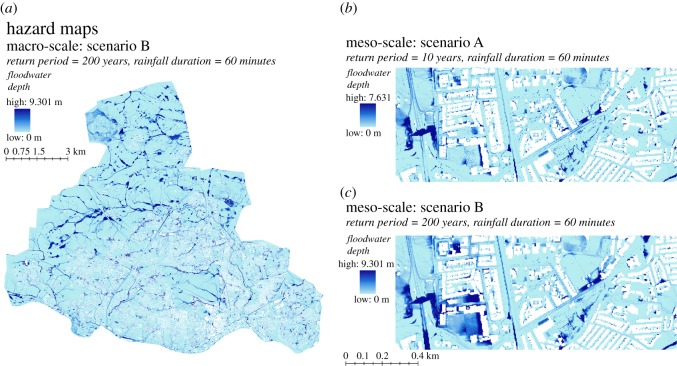
Example of a high-resolution flood simulation from the flood model for Newcastle area (UK).

A baseline undisrupted event is first run (NA_0), before the 1 in 10 and 1 in 200 year return period events (NA_10 and NA_200, respectively), both of a 1 hour duration. The two storm events were created following the standard procedure from the Flood Estimation Handbook [58]. The spatial footprints of the simulated flood event produce a time series of hazard maps showing water depths (in metres; figure 7).

**Figure 8.**
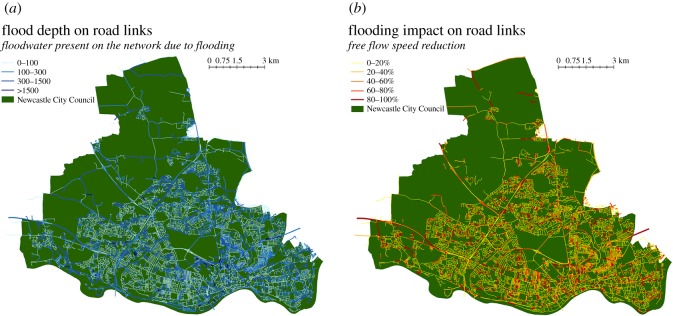
(*a*) The floodwater depth on each link of the network as a result of a 1 in 200 years flood simulation. (*b*) Impact of flooding in terms of speed reduction.

Integration of depths with the disruption function (figure 2) enables the speed reduction, according to the depth of floodwater, to be calculated for each link (figure 8). These modified network characteristics are used to recalculate traffic flows and BC pre- and post-events. The total time of delays across the whole network is considered, i.e. the sum of the delays to all journeys between all origins and destinations for journeys across Tyne and Wear region (MSOA population-weighted centroids).

### 4.2. Transport network criticality

**Figure 9.**
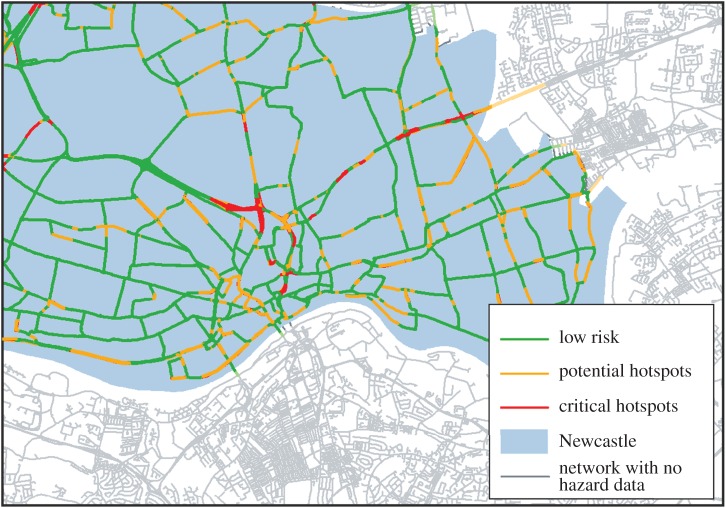
Critical hotspots in terms of both vulnerability to flooding and impact on traffic flows. Red are the most important links, green the least.

Critical links can be selected through a matrix of use and exposure [59]. Table 1 shows a risk analysis matrix that relates vulnerability to flooding with the importance of a road link, thereby providing an indication of how severely a link could be impacted by a flooding event. Figure 9 shows the application of the matrix categories to Newcastle upon Tyne to identify hotspots (in red) that are characterized by high vulnerability and high flows. This analysis highlights a small number of particularly significant links in the road network (specifically the A167, the Coast Road (A1058) and the Great North Road (B1318)).

**Table 1.** Matrix classification of vulnerability and flooding impact, highlighting indicative roads for several classifications.

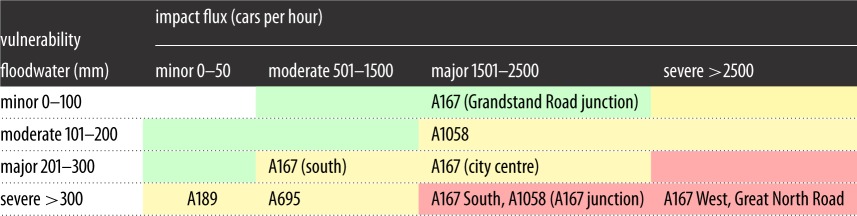

**Figure 10.**
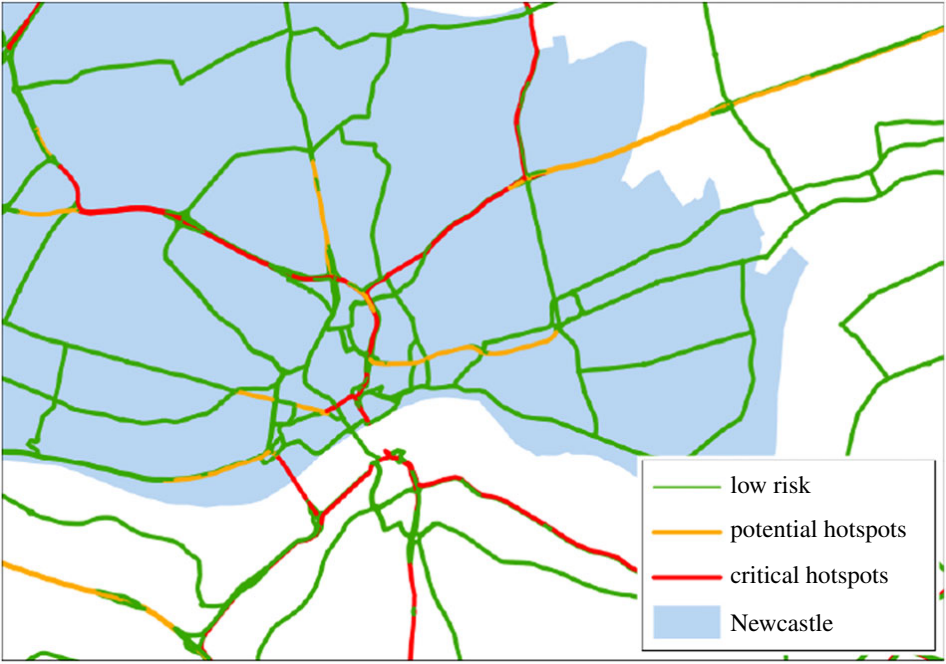
Classification of roads in Tyne and Wear based on BC of the transport network.

As an alternative and additional approach, the BC is also calculated for baseline traffic flows over the wider Tyne and Wear region and ranked, and table 2 classifies these ranks according to relative criticality. This identifies the same three locations as being critical, but also highlights the criticality of routes to the south, including the Tyne Bridge, and the A186 through the south of Newcastle city centre (figure 10).

**Table 2.** Classification of road criticality based on BC, highlighting indicative roads for several classifications.

BC range	classification	example locations
0.000–0.001 (lowest 5% of edges)	low risk	A167–A189 (slip roads between central motorway and Grandstand Road)
0.039–0.098 (second highest 5% of edges)	potential hotspots	B1318 (Great North Road)
0.098–0.193 (highest 5% of edges)	critical hotspots	A167 (central motorway)

### 4.3. Adaptation strategies

Two adaptation strategies are tested against these events to explore the effectiveness of traditional engineering (RE_10 and RE_200) approaches and a green infrastructure (GR_10 and GR_200) strategy.

**Figure 11.**
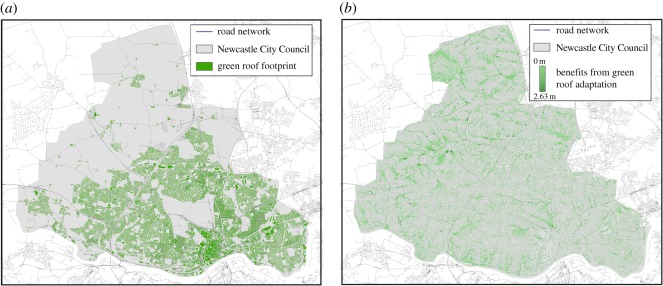
(*a*) Locations of green roofs. (*b*) The benefits from the green roof strategy in terms of reduced depth of flooding for the 1 in 200 year event.

A number of possible green infrastructure options are identified in §2.3; here each roof in the city is assumed to have the capacity to store 5 cm water depth. The roof storage delays the release of rain water onto the surface, reducing both peak flow rates and total runoff volume of rainwater compared (figure 11).

A more targeted approach is used for the traditional engineering strategy. The critical portions of the road network are used to prioritize the location of interventions. Both methods in §4.2 identify the convergence of the A167, Great North Road (B1318) and the Coast Road (A1058) as the most critical site. This location is ‘hardened’ in the simulation by making the links resistant to the 1 in 200 year flood event (i.e. floodwater cannot affect the network performance). This could be achieved in reality by (i) raising the level of the roads, (ii) improving the drainage system or (iii) installing tanks to store runoff. Each of these interventions is expensive and disruptive.

### 4.4. Discussion

The results for the baseline and six scenarios tested within the integrated assessment framework are shown in table 3. The adaptation options provide benefits in reducing the total delays both locally and globally across the whole road network. The results demonstrate the importance of understanding the structure, capacity, connectivity, flows and other attributes of the transport network.

**Table 3.** Summary of results for all scenarios analysed. The percentages are measured relative to the comparable baseline scenario.

label	return period	adaptation	average BC	standard deviation of BC	maximum BC value	total time of delay (mins)	person minute delay
NA_0	no flood	no adaptation	0.0089	0.0174	0.1926	—	—
NA_10	10 years	no adaptation	0.0094	0.0198	0.2393	63 006	783 033
NA_200	200 years	no adaptation	0.0100	0.0219	0.2527	119 115	1 438 736
RE_10	10 years	harden road junction	0.0093 (0.6%)	0.0198 (−0.4%)	0.2306 (3.5%)	58 960 (6.4%)	689 833 (11.9%)
RE_200	200 years	harden road junction	0.0096 (3.4%)	0.0214 (2.2%)	0.2500 (1.1%)	115 191 (3.2%)	1 343 612 (6.6%)
GR_10	10 years	green roof all buildings	0.0094 (0.4%)	0.0190 (2.7%)	0.2248 (6.1%)	48 965 (22.3%)	583 309 (25.5%)
GR_200	200 years	green roof all buildings	0.0096 (3.6%)	0.0206 (5.6%)	0.2525 (0.1%)	97 263 (18.3%)	118 412 (17.7%)

At the network scale, the hardening of critical links (RE_10) in the network provided only limited benefits to overall network delays, whereas the green roof strategy provided a reduction of 22.3% for the 10-year return period event and 18.3% for the 200-year event. However, when taking into account traffic flows on each route, green roofs provided reductions of 25.5% and 17.7% for the 10- and 200-year events, respectively. However, hardening of the most critical junction provided reductions of 11.9% and 6.6% for the 10- and 200-year scenarios, respectively.

The green roof strategy does provide an overall greater improvement in network performance compared with the junction hardening. However, while the green roof strategy considered here represents an absolute upper bound (i.e. 100% of all roofs) on the potential of this intervention strategy, only one hardened junction is considered and this provides a disproportionate return. Furthermore, implementing a ‘universal’ green roof strategy is unlikely to be a realistic option in an established city, at least in a short timeframe, as many roofs are unsuitable to be retrofitted at reasonable cost. Green infrastructure, however, typically provides other benefits beyond flood risk management, such as improving wellbeing, biodiversity and providing a cooling effect during heatwaves, which should be considered during urban planning.

**Figure 12.**
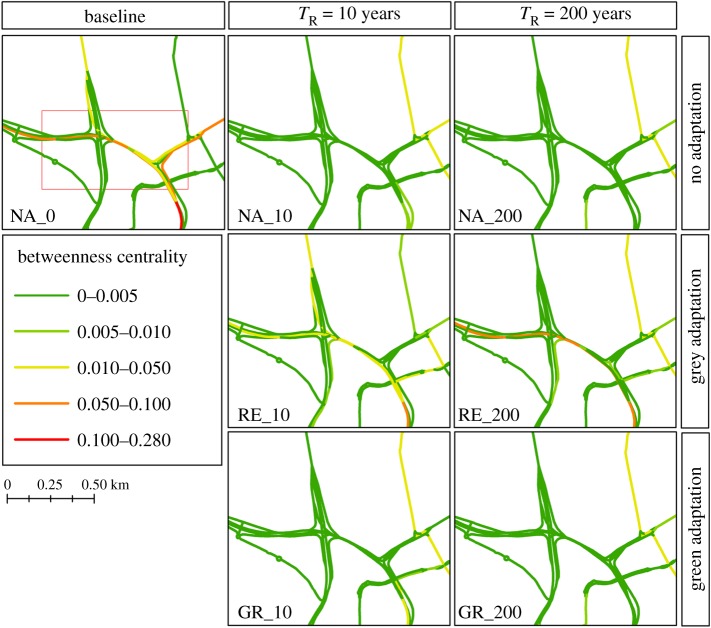
BC values for the road network around area of grey/hard adaptations for the baseline network (NA_10 and NA_200), for the grey (RE_10 and RE_200) and green (GR_10 and GR_200) adaptation scenarios for the hazard events A (1 in 10 years) and B (1 in 200 years).

Analysing the change in average and maximum BC shows reductions in BC for all adaptations. Reductions in average BC are comparable for the different adaptation strategies. However, the maximum BC is most reduced under the low return period events with a green roof strategy, but at higher return periods the hardening strategy provides the greatest reduction. Figure 12 shows the BC for the different scenarios at the location of the junction hardening strategy. Notably, the hardening strategy increases the BC values through the junction for both events, though values are still lower than the baseline. This shows that the protection of this key junction in the network increases its relative importance to the network as a whole. The effect of the green roof strategy on BC is lower than the hardening strategy because under GR_10 and GR_200 flooding can still occur at the junction causing alternative paths to be quicker. However, GR_200 has higher BC values than GR_10 because the 200-year flood has a more significant impact on other parts of the network; in particular, those in the wider Tyne and Wear region where no green roof adaptions are considered. The junction, despite being affected by flooding in the GR_200 scenario, provides a faster route than the alternatives which are not inundated in the GR_10 scenario.

The effectiveness of a single junction hardening intervention demonstrates the utility of the risk assessment matrix (table 1) that uses results from the integrated assessment modelling framework, and the calculation and ranking of the weighted BC (table 2). As the weighted BC can be calculated at lower computational cost compared with a full traffic flow analysis, it provides a rapid approach to assess and prioritize flood risk management interventions on the transport network.

While the individual components of this modelling system (such as the CityCAT flood model) have been validated separately, the validation of the combined modelling framework (using the June 2012 event) is an ongoing process. Nevertheless, the analysis offers an interdisciplinary view on a complex problem, presenting a specific indication of flood impacts on transport network and provides basis for further studies. Although the study is site-specific, data permitting, the method is readily transferable to other locations. This methodology can be applied to present conditions as well as future scenarios, allowing the examination of impacts, and appraisal of the benefits of adaptation, in the context of socio-economic and climate change. Finally, while this study focuses on the flood risk to the road network, the framework can also be applied to other weather-related phenomena and other network infrastructure (e.g. rail networks) facilitating the systematic analysis of their direct and indirect impacts.

### 4.5. Future work

The current transport model assumes people are aware of disruptions and have sufficient knowledge to identify the optimal route to minimize travel time. Non-flood disruptions (e.g. roadworks or accidents) and other uncertainties such as the influence of bad weather on journey and transport mode choice are not considered. Furthermore, while the individual components of this modelling system (such as the CityCAT flood model) have been validated separately, validation of the combined modelling framework is more complex. As shown in figure 5, transport monitoring data from the June 2012 event provide some information but this is only on a small number of routes. As monitoring technologies, and vehicle automation, become more pervasive big data analytics could provide a powerful validation tool.

This methodology will be further developed to test a wider range of flood events with different intensities and durations. Further work will also consider a broader set of adaptation options that will include other traditional, blue and green infrastructure interventions. This analysis has shown that the scale and spatial distribution of adaptation options to be important to both local and global network resilience, and a crucial development will be to consider portfolios of interventions, and their costs, rather than the extreme strategies considered here. This will provide the basis for a more comprehensive approach to appraise the benefits of adaptation of infrastructure in the urban environment. Ultimately, the methodology could be adapted to explore other hazard impacts, different types of infrastructure networks and potentially cascading failures between infrastructure systems.

## 5. Conclusion

Increased frequency of extreme flood events, coupled with population growth, require infrastructure networks to be more resilient to disruptive events. The impact of different flood events and adaptation strategies on the resilience of an urban road network has been assessed using a novel integrated assessment framework. The methodology couples simulations of hazard, traffic flows, a depth-disruption function and network analysis to measure disruption caused by floods, and the approach has been demonstrated in Newcastle upon Tyne in the northeast of England.

The results showed significant delays in travelling journeys, even for the low impact events (1 in 10 year return period). Two adaptation options (junction hardening and green roofs) were tested and both showed substantial benefits, measured in terms of reduced disruption to journeys, to network resilience during extreme events. The location of the junction to be hardened was identified by combining information on traffic flows and flood hazard, and consideration of network measures, in particular BC weighted by travel times. While the urban greening strategy provided the greatest benefits it required all roofs within the city to be reengineered, whereas a single intervention to protect a road junction from flooding provided a disproportionate benefit. The wide spatial distribution of green roofs provided a larger global benefit of 22%, but when actual traffic flows are considered the person delays are reduced by only 25%, compared with 11% from hardening a single junction. This analysis highlights that it is crucial to understand the nature of flows along, and properties of, the road network in order to prioritize key assets.

A BC measure of the network, weighted according to travel time, is shown to provide a rapid measure for prioritizing critical locations in the road network. Calculating the same metric once the network has been disrupted provides insights into the local and global benefits of different adaptation strategies across the system. Although the analysis here highlights the potential of well-targeted engineering interventions, over longer time scales adapting the urban fabric to have more green and blue infrastructure will provide systemic benefits across the city for flood risk and transport management, as well as providing other social and environmental benefits.

This framework provides a means of prioritizing limited financial resources to improve transport network resilience. This is particularly important as flood risk management investments typically have a much higher benefit cost threshold than transport infrastructure investments. In the UK, this is around 8 : 1 for transport [41] compared with 2 : 1 for flood risk management [60]. By capturing the value to the transport network from flood management interventions, it is possible to create new business models that provide benefits, and enhance the resilience of, both transport and flood risk management infrastructures.

## Data accessibility

Data supporting this publication are openly available under an ‘Open Data Commons Open Database License’. Additional metadata are available at: 10.17634/154300-16. Please contact Newcastle Research Data Service at rdm@ncl.ac.uk for access instructions. DTM, OS MasterMap and ITN are available under license from the Ordnance Survey UK. Census data available from the Office for National Statistics, UK. CityCAT is ‘property’ of Newcastle University; Python is freely available and NetworkX publicly available under a BSD license.
